# Reply Re: “Amniotic fluid from healthy term pregnancies does not harbor a detectable microbial community”

**DOI:** 10.1186/s40168-019-0640-7

**Published:** 2019-02-12

**Authors:** Efrem S. Lim, Cynthia Rodriguez, Lori R. Holtz

**Affiliations:** 1 School of Life Sciences, Arizona State University, Tempe, AZ 85287 USA; 2 Center for Fundamental and Applied Microbiomics, The Biodesign Institute, Tempe, AZ 85287 USA; 3 Department of Pediatrics, Washington University School of Medicine, 660 S. Euclid Ave., Campus Box 8208, St. Louis, MO 63110 USA

## Abstract

How and when a newborn is first colonized by microbes continues to be of great interest due to its broad implications on human health and disease. Payne et al. express their opinion about our recent study in which we characterized the virome and bacterial microbiota of amniotic fluid from 24 uncomplicated term pregnancies. We conducted additional validation studies and respond to their comments. We conclude that in amniotic fluid from healthy term pregnancies, the bacterial microbiota is indistinguishable from contamination controls, and there is no evidence of a core virome.

## Main text

How and when a newborn is first colonized by microbes continues to be of great interest due to its broad implications on human health and disease [1]. Payne et al. express their opinion about our recent study in which we characterized the virome and bacterial microbiota of amniotic fluid from 24 uncomplicated term pregnancies. We failed to identify a population of bacterial microbiota which was statistically different in concentration or content from the sequences amplified in the negative controls. Additionally, we found sparse viral reads and no evidence for a core viral community across samples.

Payne et al. comment that our findings contradict two prior studies on the amniotic fluid bacterial microbiome [2, 3]. However, these studies have important distinctions from our study of healthy term pregnancies. First, Urushiyama et al. studied amniotic fluid from women with varying degrees of placental inflammation. A major conclusion from their study is that the microbial profile in amniotic fluid of Blanc’s classification stage III chorioamnionitis may have diagnostic applications [2]. Chorioamnionitis, characterized by intrauterine inflammation or bacterial infection, has a vastly different placental histopathology and inflammatory cytokine microenvironment than healthy term pregnancies [4]. Second, Collado et al. does not include similar negative controls to assess sample extraction and preparation contamination. Third, a recent study by Rehbinder et al. demonstrated that by culture, 16S rRNA gene quantification (digital droplet), and 16S rRNA sequencing, amniotic fluid obtained from term pregnancies in which membranes are not ruptured cannot be distinguished from negative controls [5]. Therefore, taken together, maternal health likely has a major impact on the developing fetus’ first microbial exposure.

**Fig. 1:**
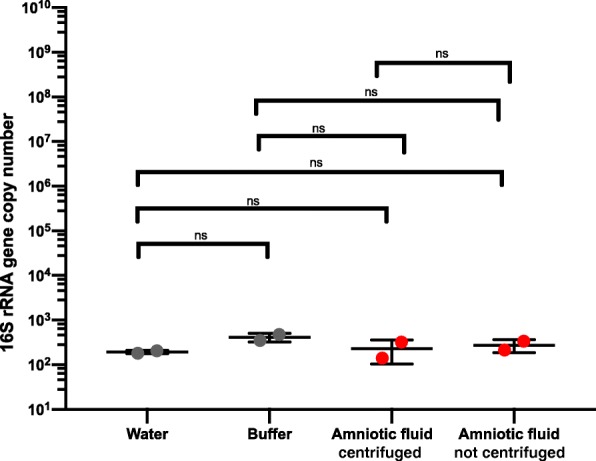
Bacterial 16S rRNA quantitative PCR. 16S rRNA gene copies per reaction were quantified in two amniotic fluid specimens in which one aliquot was centrifuged and one was not, buffer (extraction negative controls) and water (reagent negative controls). Extraction and 16S rRNA gene qPCR protocols were identical to original study [6]. Statistical significance was determined by Mann-Whitney test. ns, non-significant

On the technical merits of the study, Payne et al. argue that (A) the sample collection was biased against bacterial cells, (B) a SYBR green 16S qPCR is more appropriate, (C) the presence of human DNA may confound 16S qPCR results, and (D) samples with fewer than 5000 16S rRNA gene sequencing reads should not have been omitted. To demonstrate that the brief, low-speed centrifugation (1620 g for 5 min at 4 °C) does not bias against bacterial cells, we prospectively obtained amniotic fluid from two women with full term gestations undergoing planned C-section prior to the onset of labor. The amniotic fluid was obtained in a sterile fashion at the time of C-section by aspirating through intact amniotic membranes as described in the original study [6]. Amniotic fluid was divided into two aliquots: one aliquot was centrifuged at 1620*g* for 5 min at 4 °C (as performed in the original study [6]) and the second aliquot was not centrifuged. The amniotic fluid aliquots were subjected to the same extraction technique and 16S rRNA gene qPCR as in the original study [6]. There was no statistically significant difference in 16S rRNA copy numbers between amniotic fluid that did or did not undergo low-speed centrifugation (1620*g* for 5 min at 4 °C) (Fig. 1). The 16S rRNA gene qPCR assay performed here and in the original study [6] was indeed performed with Fast SYBR Green Master Mix (Thermo Fisher). We apologize for this error and have corrected this in a corrigendum [7]. Non-specific amplification of human DNA by “broad-range” qPCR assays indeed has been reported [8], potentially yielding over-estimates of the bacterial load in low biomass samples. We reiterate that there was no statistically significant difference between the 16S rRNA gene copy number of buffer negative controls and amniotic fluid from uncomplicated term pregnancies [6], so controlling for possible overestimation by non-specific amplification of human DNA is not needed. This illustrates the importance of appropriate negative controls in microbiome studies. Finally, sequencing depth influences the accuracy of ecological measurements [9]. The seven samples omitted from 16S rRNA gene analyses had low sequencing reads after quality filter and OTU clustering (five amniotic fluid samples, 4108, 0, 0, 159, and 1421 OTU sequences; one water control, 2439 OTU sequences; one buffer control, 1754 OTU sequences). Given the 16S rRNA gene qPCR data, we believe that this is due to the low microbial biomass of the samples. Payne et al. also argue that contamination should be relatively consistent across negative controls. Our data, however, suggest the stochastic nature of contamination and affirm the inherent challenges of sequencing low biomass samples. Thus, for these reasons, we conclude that the study was performed in accordance with microbiome best practices.

Payne et al. state that “the authors have only included OTUs in their analyses that originated from bacterial reads which were detected in the amniotic fluid samples and not in the blank extraction or PCR controls, regardless of the levels at which they were detected (Fig. 2)”. On the contrary, the data in the 16S rRNA gene analyses shown in Fig. 2a–c (OTU richness, diversity, and PCoA) of [6] includes all OTUs, not just those unique to amniotic fluid. Therefore, Fig. 2c of [6], in addition to Figure 1 of [6], shows that there is no statistically significant difference between the bacterial microbiota detected in amniotic fluid and buffer controls by either quantity or community content. As stated in the original study, subtraction of control-derived bacterial reads was only performed for the analysis shown in Fig. 2d of [6], which demonstrated the presence of low abundant rare bacterial OTUs unique to the amniotic fluid which were not frequently detected across the other amniotic fluid specimens.

In summary, our original paper [6], a recent publication from another group [5], and the data provided here converge on our original conclusions that “the most parsimonious explanation for our inability to find differences is that amniotic fluid of healthy term pregnancies has negligible bacterial biomass.” Further, “based on these analyses, we provisionally conclude that the term infant is not normally exposed to bacterial or viral populations in the immediate pre-birth interval”.
